# Supplementary material for: A fault-intrusion-tolerant system and deadline-aware algorithm for scheduling scientific workflow in the cloud
Source: PeerJ Comput Sci. 2021 Nov 2;7:e747. doi: 10.7717/peerj-cs.747 (PMC8576554; doi:10.7717/peerj-cs.747)
Supplement: Supplemental Information 6 [file peerj-cs-07-747-s006.zip › Myproject1.1/docs/org/cloudbus/cloudsim/network/TopologicalGraph.html]

TopologicalGraph


JavaScript is disabled on your browser.


- Overview
- Package
- Class
- Use
- Tree
- Deprecated
- Index
- Help

- Prev Class
- Next Class

- Frames
- No Frames

- All Classes

- Summary:
- Nested |
- Field |
- Constr |
- Method

- Detail:
- Field |
- Constr |
- Method


org.cloudbus.cloudsim.network

## Class TopologicalGraph

- java.lang.Object
- - org.cloudbus.cloudsim.network.TopologicalGraph

- ---

    

  ```
  public class TopologicalGraph
  extends java.lang.Object
  ```

  This class represents an graph containing nodes and edges, used for input with an network-layer
  Graphical-Output Restricions! EdgeColors: GraphicalProperties.getColorEdge NodeColors:
  GraphicalProperties.getColorNode

  Since:
  :   CloudSim Toolkit 1.0

- - ### Constructor Summary

    Constructors

    Constructor and Description

    `TopologicalGraph()`

    just the constructor to create an empty graph-object
  - ### Method Summary

    Methods

    | Modifier and Type | Method and Description |
    | --- | --- |
    | `void` | `addLink(TopologicalLink edge)` adds an link between two topological nodes |
    | `void` | `addNode(TopologicalNode node)` adds an Topological Node to this graph |
    | `java.util.Iterator<TopologicalLink>` | `getLinkIterator()` return an iterator through all network-graph links |
    | `java.util.Iterator<TopologicalNode>` | `getNodeIterator()` returns an iterator through all network-graph nodes |
    | `int` | `getNumberOfLinks()` returns the number of links contained inside the topological-graph |
    | `int` | `getNumberOfNodes()` returns the number of nodes contained inside the topological-graph |
    | `java.lang.String` | `toString()` prints out all internal node and link information |

    - ### Methods inherited from class java.lang.Object

      `clone, equals, finalize, getClass, hashCode, notify, notifyAll, wait, wait, wait`

- - ### Constructor Detail


    - #### TopologicalGraph

      ```
      public TopologicalGraph()
      ```

      just the constructor to create an empty graph-object
  - ### Method Detail


    - #### addLink

      ```
      public void addLink(TopologicalLink edge)
      ```

      adds an link between two topological nodes

      Parameters:
      :   `edge` - the topological link


    - #### addNode

      ```
      public void addNode(TopologicalNode node)
      ```

      adds an Topological Node to this graph

      Parameters:
      :   `node` - the topological node to add


    - #### getNumberOfNodes

      ```
      public int getNumberOfNodes()
      ```

      returns the number of nodes contained inside the topological-graph

      Returns:
      :   number of nodes


    - #### getNumberOfLinks

      ```
      public int getNumberOfLinks()
      ```

      returns the number of links contained inside the topological-graph

      Returns:
      :   number of links


    - #### getLinkIterator

      ```
      public java.util.Iterator<TopologicalLink> getLinkIterator()
      ```

      return an iterator through all network-graph links

      Returns:
      :   the iterator throug all links


    - #### getNodeIterator

      ```
      public java.util.Iterator<TopologicalNode> getNodeIterator()
      ```

      returns an iterator through all network-graph nodes

      Returns:
      :   the iterator through all nodes


    - #### toString

      ```
      public java.lang.String toString()
      ```

      prints out all internal node and link information

      **Overrides:**
      :   `toString` in class `java.lang.Object`


- Overview
- Package
- Class
- Use
- Tree
- Deprecated
- Index
- Help

- Prev Class
- Next Class

- Frames
- No Frames

- All Classes

- Summary:
- Nested |
- Field |
- Constr |
- Method

- Detail:
- Field |
- Constr |
- Method
